# Functional Analysis of *atfA* Gene to Stress Response in Pathogenic Thermal Dimorphic Fungus *Penicillium marneffei*

**DOI:** 10.1371/journal.pone.0111200

**Published:** 2014-11-03

**Authors:** Panjaphorn Nimmanee, Patrick C. Y. Woo, Pramote Vanittanakom, Sirida Youngchim, Nongnuch Vanittanakom

**Affiliations:** 1 Department of Microbiology, Faculty of Medicine, Chiang Mai University, Chiang Mai, Thailand; 2 State Key Laboratory of Emerging Infectious Diseases, Research Centre of Infection and Immunology and Carol Yu Centre for Infection, The University of Hong Kong, Hong Kong, China; 3 Faculty of Medicine, University of Phayao, Phayao, Thailand; University of Wisconsin - Madison, United States of America

## Abstract

*Penicillium marneffei*, the pathogenic thermal dimorphic fungus is a causative agent of a fatal systemic disease, penicilliosis marneffei, in immunocompromised patients especially HIV patients. For growth and survival, this fungus has to adapt to environmental stresses outside and inside host cells and this adaptation requires stress signaling pathways and regulation of gene expression under various kinds of stresses. In this report, *P. marneffei* activating transcription factor (*atfA*) gene encoding bZip-type transcription factor was characterized. To determine functions of this gene, *atfA* isogenic mutant strain was constructed using the modified split marker recombination method. The phenotypes and susceptibility to varieties of stresses including osmotic, oxidative, heat, UV, cell wall and cell membrane stresses of the mutant strain were compared with the wild type and the *atfA* complemented strains. Results demonstrated that the mRNA expression level of *P. marneffei atfA* gene increased under heat stress at 42°C. The *atfA* mutant was more sensitive to sodium dodecyl sulphate, amphotericin B and *tert*-butyl hydroperoxide than the wild type and complemented strains but not hydrogen peroxide, menadione, NaCl, sorbitol, calcofluor white, itraconazole, UV stresses and heat stress at 39°C. In addition, recovery of *atfA* mutant conidia after mouse and human macrophage infections was significantly decreased compared to those of wild type and complemented strains. These results indicated that the *atfA* gene was required by *P. marneffei* under specific stress conditions and might be necessary for fighting against host immune cells during the initiation of infection.

## Introduction

*Penicillium marneffei* (has been combined in Genus *Talaromyces* based on new molecular phylogenetic analysis [1]) is a pathogenic fungus that causes a fatal systemic mycosis in HIV-positive persons, patients with systemic lupus erythematosus (SLE) and patients who receive immunosuppressive drug during organ transplantation [2], [3]. Unlike other *Penicillium* spp., *P. marneffei* is a dimorphic fungus that possesses two distinct cellular forms regulated by temperature. At 25°C, this fungus grows as mycelia and produces conidia, whereas, at 37°C, it grows as a yeast-like cell dividing by fission [2], [4]. Humans acquire *P. marneffei* via inhalation of fungal conidia into the lungs. Once inside the host, *P. marneffei* is able to multiply inside alveolar macrophages as fission yeast cells and disseminates throughout the host body by the hematogenous route [5], [6]. For pathogenic fungi, if they are unable to overcome the host defensive mechanisms, especially reactive oxygen species (ROS) produced by host immune cells and other stresses inside the host microenvironment, they cannot establish the disease and will be eliminated from the host body [7]. However, the systems that regulate the survival of *P. marneffei* under various stresses outside and inside host cells are still unclear.

Two component signaling systems are common signal transduction strategies found in both prokaryotes and eukaryotes using in response to environmental signals [8]. In fungi, these systems include multi-step phosphorelay proteins, a sensor histidine kinase protein, a histidine-containing phosphotransfer (HPt) protein and a response regulator protein [9]. In unstressed cells of *Saccharomyces cerevisiae*, there is an autophosphorylation on a histidine residue in a membrane-bound sensor kinase, Sln1. The phosphate is transferred to an aspartate residue on the receiver domain of the same protein and is subsequently transferred to a histidine residue in an HPt protein, Ypd1. Phosphorylated Ypd1 transfers phosphate to a response regulator, Skn7 or Ssk1 [9]–[11]. *S. cerevisiae* Skn7 is a response regulator that also acts as transcription factor and plays a role in antioxidation and cell-wall biosynthesis regulation [9]. In pathogenic fungi, *Candida albicans*, *Cryptococcus neoformans* and *Aspergillus fumigatus*, Skn7 functions in adaptation to oxidative stress and contribute to their virulence [9], [12]. For Ssk1, under osmotic or oxidative stress, there is no phosphotransfer through Sln1-Ypd1-Ssk1 proteins. Unphosphorylated Ssk1 can activate the Hog1 MAPK pathway by binding to the MEKK protein, Ssk2. After activation, phosphorylated Hog1 translocates from cytosol to the nucleus and regulates transcriptions of genes involved in stress adaptation. In fission yeast *Schizosaccharomyces pombe*, the Sty1 pathway which is an Hog1 pathway homolog plays a role in a global stress response [13]. Under stress conditions, Sty1 translocates to the nucleus and phosphorylates the transcription factor Atf1 both in vitro and in vivo. Atf1, homolog of mammalian ATF2, is a basic-region leucine zipper (bZip)-type transcription factor that binds to the CRE sequence (T[G/T]ACGT[C/A]A) of the target genes in response to stress [8], [14]. In filamentous fungus, *Aspergillus nidulans*, stress activated kinase A, SakA (Hog1 homolog) translocates to the nucleus to interact with AtfA (Atf1 homolog) in response to oxidative or osmotic stress signal and AtfA also plays a role in oxidative and heat stress responses on conidia [8], [15]. For pathogenic dimorphic fungus *P. marneffei*, it has been shown that Skn7 encoding gene is involved in oxidative stress response [16]. However, signal transductions under stress condition throughout the stress activated kinase (SAPK) pathway or SakA and AtfA transcription factor are not well understood.

We have identified the *P. marneffei sakA* gene and proposed that this gene participated in asexual development, yeast cell production in vitro and inside macrophages, oxidative and heat stress responses and chitin deposition along the hyphae of *P. marneffei* (unpublished data). In this study, *P. marneffei* transcription factor gene, *atfA* was isolated and the *atfA* mutant was constructed to characterize the role of this gene under stress conditions. The results demonstrated that *P. marneffei atfA* gene encoded protein containing conserved bZip domain found in a family of bZip transcription factors and this gene is partly involved in viability under oxidative stress but not osmotic, UV and heat stresses. In addition, this gene is also required for survival of *P. marneffei* inside host macrophages.

## Materials and Methods

### Fungal strains and culture conditions

*P. marneffei* (CBS 119456) was obtained from an AIDS patient from the Central Laboratory, Maharaj Nakorn Chiang Mai Hospital, Thailand in 1999 [17]. The fungus was grown on potato dextrose agar (PDA) (Difco Becton Dickinson and Company, NJ USA) or malt extract agar (MEA) (OXOID Hampshire England) for seven days at 25°C. The *sakA* and *atfA* mutants generated from this isolate were maintained on media containing 200 µg/ml of hygromycin. The *sakA* and *atfA* complemented strains were maintained on media containing both 200 µg/ml hygromycin and two µg/ml bleomycin (Sigma-Aldrich, St. Louis USA). For long-term storage, mycelia of given strains were suspended in 30% (w/v) sterile glycerol and frozen at −70°C. Conidial suspension was prepared as previously described [18]. Briefly, following the scraping of the colony surface with a cotton swab and the suspension of mycelia in sterile 0.01% Tween 80, conidia were then isolated from the mycelia by filtration through sterile glass wool.

### Molecular biology procedures and plasmid constructions

#### *atfA* sequencing and sequence analysis

The complete genomic sequence of the *atfA* gene was obtained by PCR amplification using the genomic DNA of *P. marneffei* strain F4 as the DNA template. Primers AtfA-WF and AtfA-WR (Table 1) were designed based on the genome database of *P. marneffei* ATCC 18224 (whole genome shotgun sequencing project; http://www.ncbi.nlm.nih.gov). The 1677-bp amplicon was sequenced in both directions. The NCBI BLAST program (http://www.ncbi.nlm.nih.gov) was used to search for nucleotide and protein sequence similarities. The programs ‘nucleic acid translation’ of BioEdit Sequence Alignment Editor Software was used to predict an open reading frame and deduced amino acid sequences from the nucleotide sequences. Conserved domains of AtfA putative protein were predicted using ScanProsite tool (http://prosite.expasy.org/scanprosite/). Deduced amino acid sequences of the *atfA* genes of other fungal homologous sequences that were obtained from GenBank databases (http://www.ncbi.nlm.nih.gov) were used for multiple alignments. Multiple sequence alignment was generated with the ClustalW program (http://www. ebi.ac.uk/clustalw/index.html).

**Table 1:** PCR primers used in this study.

Primer name	Sequence (5′ to 3′)	Reference
AtfA-A1	AGGAACGTACCACCACTGAA	This study
5′ atfARev500	CCAGCATAGCAGGACTCAGC	This study
AtfA-A3	CGTTACCCAACTTAATCGCCTTGCGTACAACCTCGCAACCAAT	This study and [19]
AtfA-A4	GTGTCATGTCCAGTCGAGTCC	This study
HY	GGATGCCTCCGCTCGAAGTA	[19]
YG	CGTTGCAAGACCTGCCTGAA	[19]
AtfAF-RT	CGCTGAGTCCTGCTATGCTG	This study
AtfAR-RT	GCTCGACCTTGGCTTGGAGA	This study
AtfA-WF	GCCATGACCTCACAATTACC	This study
AtfA-WR	ATTGGTTGCGAGGTTGTACG	This study
AtfA-ComF	GTGCGAAGCTT *GTGTCGAATTGGCCATGTTG	This study
AtfA-ComR	CTATAGGTACC **GACAAGGCATCGTCGACAC	This study
Pm1	ATGGGCCTTTCTTTCTGGG	[23]
Pm2	GCGGGTCATCATAGAAACC	[23]
LPW21406_GAPDH	TGGTCTAGCTCGAATCCAAG	[25]
LPW21407_GAPDH	GTCGACGTAGGCCTCAGTTA	[25]
atfA_exon2	CCGAAGAAGATGACTGATGAAG	This study
atfA_exon3	TTGCAAGCCACTGCTTCTTA	This study

*HindIII restriction site sequence,

**KpnI restriction site sequence.

#### Disruption and complementation of *atfA*

**Figure 1:**
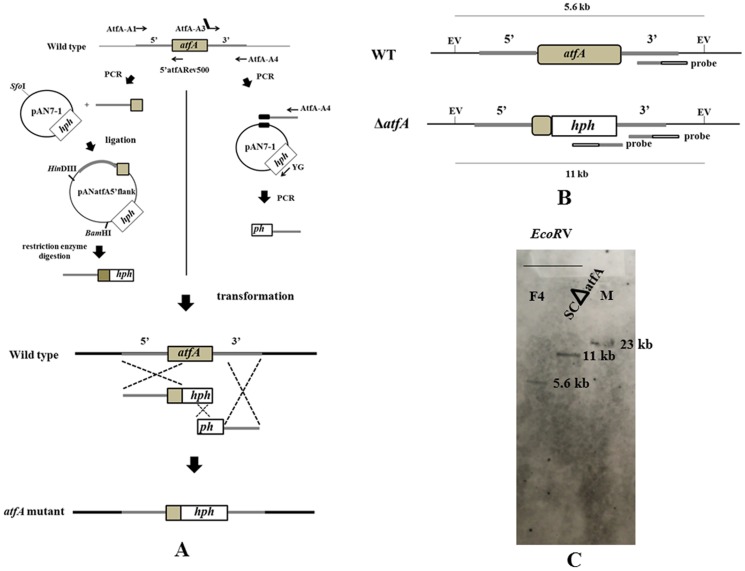
Strategy for deletion of the *atfA* gene by replacing the entire *atfA* ORF with two DNA fragments using modified split marker method. (A) For the first DNA fragment, PCR amplification of 500 nucleotides of the *atfA* gene with 5′ flanking region from genomic DNA of wild type is performed. The PCR product is ligated to pAN7-1 containing the *hph* gene. The DNA fragment containing 500 nucleotides of the *atfA* gene with 5′ flanking region and the *hph* gene without terminator (*hph*) is obtained by digestion of recombinant plasmid with *Hin*dIII and *Bam*HI. For the second fragment, 3′ *atfA* flanking region of *atfA* is amplified and PCR product is used as a template with pAN7-1 in the second round PCR. Product form this PCR step consists of 3′ *atfA* flanking region and the truncated sequence of the *hph* gene (*ph*) with terminator. Two DNA fragments are then transformed into *P. marneffei* wild type to generate *atfA* mutant strain. The primers used for mutant construction and the predicted results of three homologous recombinations of 5′ and 3′ *atfA* flanking regions and *hph* gene at the *P. marneffei atfA* locus in split marker recombination method are shown (B) The restriction map demonstrates recognition sites of *Eco*RV (EV) used in Southern blot analysis to detect the deletion of *atfA* gene in the *atfA* mutant strain (Δ*atfA*) comparing to the wild type strain (WT). The positions of probe that is specific to both 3′ flanking region of *atfA* gene (grey bar) and *hph* gene (empty bar) are identified. EV represents *Eco*RV. (C) Result of Southern blot hybridization. Probe containing 0.8 kb fragment of 3′ flanking region of *atfA* gene and 1.5 kb fragment of *hph* gene hybridized with 5.6 kb fragments of *Eco*RV-digested DNA from the wild type strain and hybridized with a 11 kb fragment of *Eco*RV-digested DNA from the *atfA* mutant strain.

To disrupt the *atfA* gene, the modified split marker recombination approach was used [19]. Briefly, primers AtfA-A1 and 5′ atfARev500 and primers AtfA-A3 and AtfA-A4 (Table 1) were designed for generating two DNA fragments (Figure 1A). The first fragment generated by the AtfA-A1 and 5′ atfARev500 contained 5′ flanking region and approximately 500 bp of the *atfA* gene, whereas the second fragment generated by AtfA-A3 and AtfA-A4 included 3′ flanking sequences fused to the incomplete sequences of plasmid pAN7-1 [20] that contained the hygromycin resistance (*hph*) gene. The first fragment (1.9-kb amplicon) was cloned into *Sfo*I site of pAN7-1 plasmid to give pANatfA5′ flank and the second fragment was used as template for the second round PCR with pAN7-1 using primers AtfA-A4 and YG (Table 1) to generate 3.1-kb fragment containing truncated fragment of the *hph* gene and 3′ flanking region of the *atfA* gene. *P. marneffei* protoplasts were transformed with 2–5 µg of the *Hin*dIII-*Bam*HI fragment from pANatfA5′ flank containing 5′ flanking region with 500 bp of the *atfA* gene and the *hph* gene and the 3.1-kb fragment generated by primers AtfA-A4 and YG. The *atfA* mutants were screened on brain heart infusion agar (BHA) (OXOID Hampshire England) containing 200 µg/ml hygromycin (Sigma-Aldrich, St. Louis USA) and the selected mutants were confirmed by PCR and Southern blot analysis. To ensure a complete absence of *atfA* transcript, RT-PCR was performed using primers AtfAF-RT and AtfAR-RT (Table 1).

**Figure 2:**
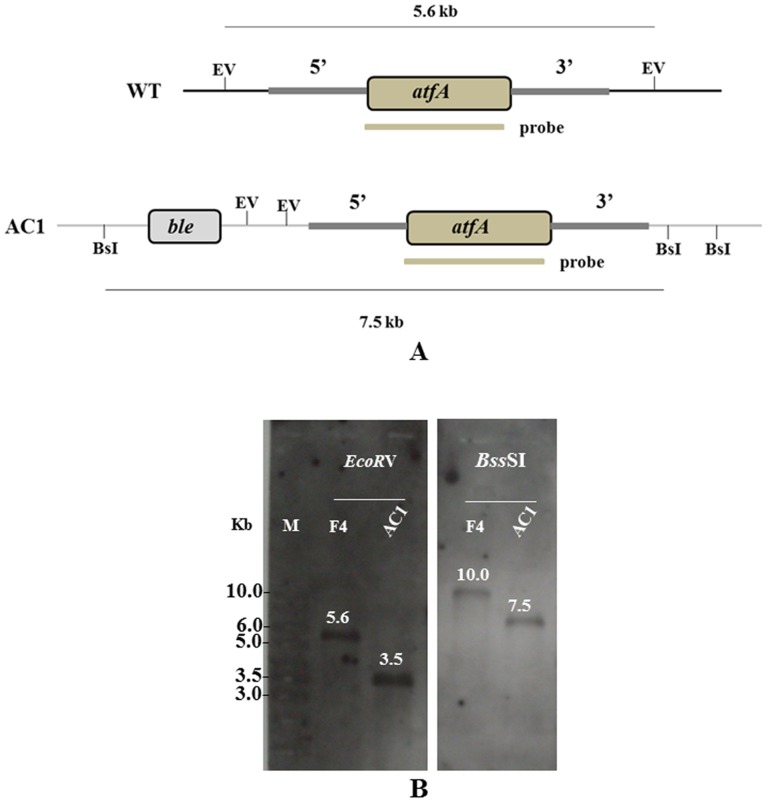
Strategy for construction of the *atfA* complemented strain. (A) The restriction map demonstrates recognition sites of *Eco*RV (EV) and *Bss*SI (BsI) used in Southern blot analysis to detect the presence of *atfA* gene in the *atfA* complemented strain (AC1) comparing to the wild type strain (WT). The grey bar indicates the position of probe that is specific to the *atfA* gene. (B) Southern blot hybridization of genomic DNA from the wild type (F4) and the *atfA*-complemented (AC1) strains using probe in Figure 2A. Probe (a 0.9 kb fragment of *atfA*) hybridized with 5.6 kb fragment and unpredicted fragment (10.0 kb fragment) of *Eco*RV- and BssSI-digested DNA from the wild type strain, respectively. For the *atfA* complemented strain, probe hybridized with unpredicted fragment (3.5 kb fragment) and 7.5 kb fragment of *Eco*RV- and BssSI-digested DNA from the *atfA* complemented strain, respectively.

The *atfA* complementation construct was generated by amplification of the *atfA* coding region plus 2.5 kb of promoter and 1.7 kb of 3′ flanking region using primers AtfA-ComF containing *Hin*dIII site and AtfA-ComR containing *Kpn*I site (Table 1). The 5.7-kb PCR product was digested with *Hin*dIII/*Kpn*I and ligated into *Hin*dIII/*Kpn*I digested pJL43b1 [21] that contained bleomycin resistance gene (*ble*) generating pJLatfA. The plasmid pJLatfA was transformed into the *atfA* mutant strain using protoplast transformation method. After transformation, the complemented strains were screened on BHA containing both 200 µg/ml hygromycin and 2 µg/ml bleomycin (Sigma-Aldrich, St. Louis USA). The selected strains were confirmed by using PCR amplification using primers AtfAF-RT and AtfAR-RT (Table 1) and Southern hybridization (Figure 2A).

#### Expression analysis

RNA was isolated from vegetative hyphal cells of *P. marneffei* wild type strain grown at 25°C for three days in Sabouraud dextrose broth (SDB) (Difco Becton Dickinson and Company, NJ USA), from asexual developing conidia collected from cultures grown on PDA at 25°C for seven days and from yeast cells grown in brain heart infusion (BHI) (OXOID Hampshire England) at 37°C for six days [22]. For expression under stress conditions, RNA was isolated from conidia, mycelia and yeast cells of *P. marneffei* wild type strain that were incubated at 39°C or were added to one mM hydrogen peroxide (H_2_O_2_) for one hour. The RNA was extracted using NucleoSpin RNA II (MACHEREY-NAGEL, GmbH & Co.KG Düren, Germany). RNA was treated with rDNase according to the manufacturer's instructions prior to RT-PCR analysis and a no cDNA synthesis control was performed to ensure for non DNA contamination. cDNA synthesis was performed using a Thermo Scientific RevertAid First Strand cDNA Synthesis Kit (Fermentas, Burlington, Canada). 18S rRNA was amplified using the primers Pm1 and Pm2 ([23], Table 1) and used as a loading control. Expression of *sakA* was determined using the primers AtfAF-RT and AtfAR-RT (Table 1).

For real time PCR, RNA was extracted from conidia of *P. marneffei* wild type strain that were incubated at 25°C or 42°C, 250 rpm for 0, 10, 20, 30 and 40 minutes. cDNA synthesis was performed and the samples were amplified in reaction mixtures containing FastStart DNA Master SYBR Green I Mix reagent kit (Roche, Basel, Switzerland) using a ABI 7900 HT Fast Real-Time PCR System (Applied Biosystems, Foster City, CA, USA). Real-time PCR was performed using standard qPCR conditions [24] including 40 cycles of 95°C for 15 s, followed by 60°C for one minute and dissociation curve (95°C for 15 s, followed by 60°C for 15 s and 95°C for 15 s) in the control software of SDS 2.4 (Applied Biosystems, Foster City, CA, USA). Primers atfA_exon2 and atfA_exon3 (Table 1) were designed and used for *atfA* gene expression. Glutaraldehyde-3-phosphate dehydrogenase (GAPDH) gene were amplified using primers LPW21406_GAPDH and LPW21407_GAPDH ([25], Table 1) and gene expression levels of this gene were used to normalize the amounts of input RNA. The relative quantitative expression levels were calculated using the 2^−ΔΔC^_T_ method [26]. Three independent experiments were performed and unpaired *t*-test (http://graphpad.com/quickcalcs/ttest1.cfm?Format=SD) was used for data analysis.

### Phenotypes of *P. marneffei*

Morphologies of *P. marneffei* wild type and the *atfA* mutant were characterized under the microscope using slide culture technique on PDA incubated at 25°C for four, seven and ten days. To visualize chitin deposition and cell wall, fungi were stained with calcofluor white (CFW) and observed under a fluorescence microscope (Nikon Eclipse 50i, Tokyo, Japan).

For yeast cell induction at 37°C, conidia of wild type and the *atfA* mutant were inoculated on Sabouraud dextrose agar (SDA) and in SDB and incubated at 37°C for ten days and six days, respectively. Yeast cell morphologies were visualized under a microscope (Nikon Eclipse 50i Tokyo, Japan).

### Stress susceptibility of *P. marneffei atfA* deletion mutant

In osmotic, oxidative, cell wall and cell membrane stress susceptibility studies of conidia, the drop dilution assay was used. Conidia of *P. marneffei* wild type, *atfA* mutant and *atfA* complemented strains were isolated and were counted using a hemocytometer chamber. For drop dilution assay, series of ten-fold dilutions derived from a starting solution of 1×10^7^ conidia/ml to 1×10^3^ conidia/ml were spotted in aliquots of five microliters onto minimal medium (MM) plates [27] supplemented with/without sorbitol, NaCl, H_2_O_2_, *tert*-butylhydroperoxide (*t*-BOOH), menadione (Md), CFW, sodium dodecyl sulphate (SDS), amphotericin B and itraconazole and then incubated at 25°C or 37°C for five days.

For heat stress condition, conidia from wild type, *atfA* mutant and *atfA* complemented strains were collected and drop dilution assay was performed on MM agar plates at 39°C for five days. For viability at 42°C, conidia of each strain were inoculated into BHI broth and incubated at 42°C, 250 rpm. After one hour, conidia were diluted and plated on SDA for colony forming unit count. A number of colonies on control plate at 25°C were used as the baseline for calculation of % survival at 42°C.

UV susceptibility was done as previously described [28]. Approximately one hundred conidia of wild type, *sakA* mutant and *atfA* mutant were spread on SDA plates and were exposed under different doses of UV light (254 nm) including 0, 2000, 4000, 6000 and 8000 microjoules/cm^2^ using CL-1000 Ultraviolet crosslinker (UVP, Upland, CA, USA). Plates were incubated at 25°C for three to four days and colony forming units (CFUs) on plate zero microjoules/cm^2^ were used as the baseline values for calculating the percentage survival of conidia at different UV doses.

All stress susceptibility experiments were performed in triplicate.

### Survival of *P. marneffei* inside macrophages

To investigate survival of *P. marneffei atfA* mutant inside macrophages, the intracellular survival assays were done as previously described [3]. J774 mouse monocyte macrophages (Sigma-Aldrich, St. Louis USA) were maintained in Dulbecco's Modified Eagle Medium: DMEM (Gibco-life technologies, New York USA) supplemented with 10% fetal bovine serum and THP-1 human monocytes (American type culture collection, ATCC) were grown in RPMI-1640 medium (Gibco-life technologies, New York USA) containing 10% fetal bovine serum at 37°C, 5% CO_2_.

For infection, J774 macrophages were seeded into a 24-well tissue culture plate (TPP, Trasadingen, Switzerland) at a concentration of 4×10^5^ cells per well and incubated at 37°C, 5% CO_2_ for 24 hours before adding fungal conidia. THP-1 monocytes were seeded to a 24-well tissue culture plate at a concentration of 1×10^6^ cells/well and allowed to differentiate to macrophages in RPMI supplemented with 100 nM phorbol 12-myristate 13-acetate (PMA) and incubated at 37°C, 5% CO_2_ for 72 hours. After incubation, culture media were replaced with fresh culture media containing conidia of wild type, *atfA* mutant and *atfA* complement strains at a concentration of 1×10^6^ cells/well (multiplicity of infection: MOI of 2.5 for J774 and MOI of 1 for THP-1). Cells were incubated for two hours to allow adhesion and phagocytosis of the conidia. After two hours, each well was washed with media containing 240 U/ml of nystatin (Sigma-Aldrich, St. Louis USA) to kill extracellular conidia. Nystatin was replaced by fresh media and incubated for 24 hours. After incubation, infected macrophages were lysed with 1% Triton X-100 (Sigma-Aldrich, St. Louis USA). Cell lysates were diluted and plated on SDA and incubated at 25°C for colony forming unit (CFU) count. The CFUs harvested from cell lysates at two hours were used as the initial inocula that acted as the baseline values for intracellular survival analysis. CFUs harvested at 24 hours were used for calculation of the percentage recovery of fungal conidia inside macrophages. The experiments were performed in triplicates and analyzed using standard *t*-tests (http://www.graphpad.com/quickcalcs/ttest1.cfm?Format=SD).

### Nucleotide sequence accession number

The nucleotide sequence of the *atfA* gene was submitted to the GenBank database under accession number KF636750.

## Results

### *P. marneffei atfA* encodes a putative bZip transcription factor

The *atfA* gene of *P. marneffei* strain F4 has 1,536 nucleotides, with three introns. The 1,230 nucleotide mRNA predicted a 409-amino-acid protein with a molecular mass of 43.5 kDa with high similarity to bZip transcription factor. This protein revealed 99.76% identical to the analogous *P. marneffei* ATCC 18224 (EEA27441), 67.24% identical to *A. fumigatus* Af293 AtfA (EAL92448), 65.77% identical to *A. oryzae* RIB40 AtfA (XP_001819834), 65.04% identical to AtfA of *A. nidulans* FGSC A4 (CBF83765) and 29.83% identical to Atf1 of *S*. *pombe* 972h- (NP_595652). The conserved basic-leucine zipper (bZip) domain found in the bZip transcription factor family was shown at amino acid 352–405. This suggested that *P. marneffei atfA* gene encoded a member of the putative bZip transcription factor.

**Figure 3:**
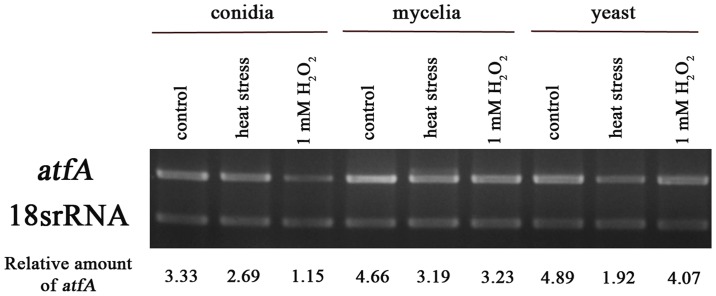
*atfA* expression during phase transition. RNA was isolated from *P. marneffei* strain F4 cells including conidia collected from cultures grown for seven days on PDA at 25°C, three days in SDB at 25°C (mycelia), and six days in BHI broth at 37°C (yeast). 18S rRNA was used as loading control of each growth phase.

To investigate the expression of *atfA* in *P. marneffei*, RNA was isolated from wild type vegetative hyphae grown for three days in SDB at 25°C, asexual development (conidiation) collected form cultures grown for seven days on PDA at 25°C and yeast cells grown for six days in BHI broth at 37°C. The *atfA* transcript was detected by reverse transcriptase (RT)-PCR. Comparing with 18S rRNA loading control, *atfA* transcript determined during asexual development (conidia) was less than those cells during vegetative hyphal growth (mycelia) at 25°C and during yeast growth at 37°C, respectively (Figure 2). The amount of *atfA* transcript was not increased in conidia, mycelia and yeast under both heat shock at 39°C and oxidative stress with one mM H_2_O_2_ (Figure 3).

### *atfA* deletion does not affect asexual development and yeast cell production

To determine the functions of the *atfA* gene in *P. marneffei*, the *atfA* mutant strain was constructed by replacing the open reading frame of the *atfA* gene with the *hph* cassette using modified split marker method (Figure 1A). The transformant lacking the *atfA* gene was screened by PCR using primers specific to *atfA* and *hph* genes. Five clones were selected and the result from Southern blot hybridization showed that one clone, denoted SCΔatfA, contained a single copy of the *hph* gene integrated within the *atfA* gene (Figure 1B and 1C).

To confirm the function of the *atfA* gene, *atfA* complemented strains were constructed. Plasmid containing promoter, *atfA* coding sequence and 3′ region of *atfA* was transformed into the *atfA* mutant strain (SCΔ*atfA*). After transformation, three clones including AC1, AC2, and AC3 were selected and PCR amplification using primers AtfAF-RT and AtfAR-RT (Table 1) demonstrated that all clones contained *atfA* gene. However, Southern blot analysis showed that only AC1 revealed a single copy of the *atfA* gene (Figure 2A and 2B) and this clone was used in further studies.

**Figure 4:**
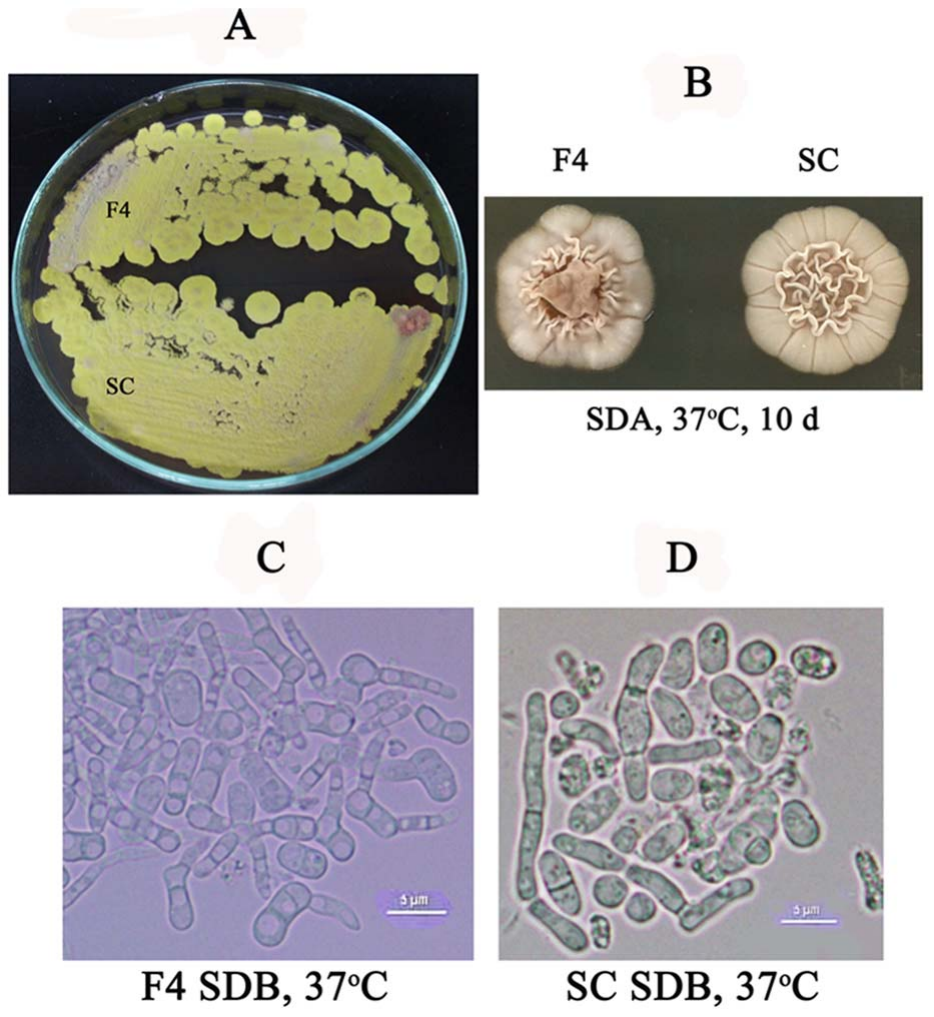
Morphology of *P. marneffei atfA* mutant compared with wild type strain. (A) Colonies of wild type (F4) and *atfA* mutant (SC) on PDA incubated at 25°C for seven days. (B to D) Conidia isolated from wild type (F4) and *atfA* mutant (SC) were inoculated on SDA and SDB and were incubated at 37°C for ten days and six days, respectively. Scale bar represents five micrometers.

To investigate colony morphologies, conidia of the wild type and the mutant strains were inoculated on PDA and SDA and the plates were incubated at 25°C and 37°C, respectively. The result showed that *atfA* mutant strain had colony morphology similar to those of the wild type strain at both temperatures (Figure 4A, 4B). In addition, yeast cell production of the *atfA* mutant was also undistinguishable from the wild type strain (Figure 4C, 4D).

### *atfA* gene participates in oxidative but not osmotic stress responses of *P. marneffei* conidia

**Figure 5:**
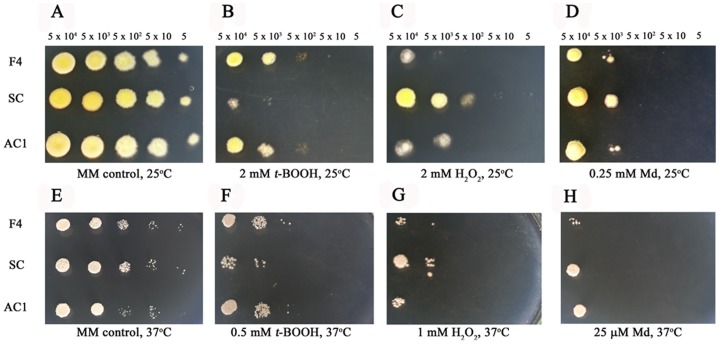
Susceptibility to oxidative stresses of *P. marneffei*. Growth of *P. marneffei* wild type (F4), the *atfA* mutant (SC) and *atfA* complemented strain (AC1) at 25°C and 37°C on MM agar supplemented with 2 and 0.5 mM *t*-BOOH (B and F), 2 and 1 mM H_2_O_2_ (C and G), and 0.25 mM and 25 µM menadione (D and H). Five microliters of cell dilutions (5×10^4^ to 5 cells) were inoculated on MM agar containing each compound. (A) and (E) represent MM control plates at 25°C and 37°C, respectively.

To determine the function of the *atfA* gene on stress response, the growth of wild type, *atfA* mutant and *atfA* complemented strains on media supplemented with or without different stressors were evaluated. For oxidative stress at 25°C, *atfA* mutant strain was more slightly sensitive to two mM *t*-BOOH (Figure 5B) comparing to the wild type and complemented strains. However, growths of all strains were undistinguished under stresses from both two mM H_2_O_2_ and 0.25 mM menadione (Figure 5C and 5D). At 37°C, a slightly higher susceptibility to *t*-BOOH (0.5 mM) was observed in the mutant (Figure 5F) and no difference among the mutant, wild type, and complemented strains under one mM H_2_O_2_ and 25 µM menadione stresses (Figure 5G, 5H).

**Figure 6:**
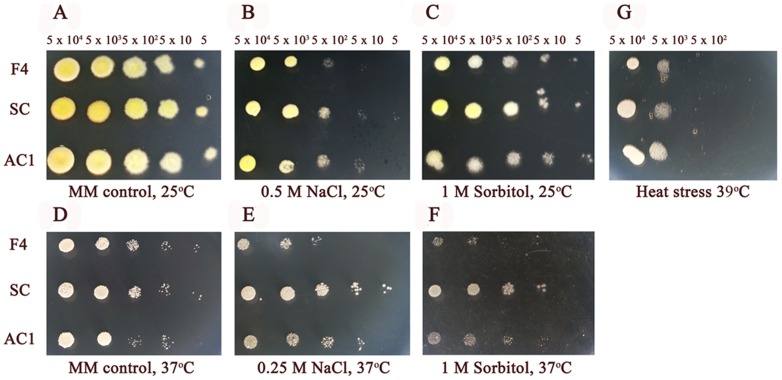
*atfA* gene is not involved in osmotic and heat stress responses in *P. marneffei*. Five microliters of cell dilutions (5×10^4^ to 5 cells) of wild type (F4), *atfA* mutant (SC) and *atfA* complemented (AC1) strains were inoculated on MM agar supplemented with 0.5 and 0.25 M NaCl (B and E) and 1 M sorbitol (C and F). (G) MM agar was incubated at 39°C. (A) and (D) represent MM control plates at 25°C and 37°C, respectively.

For osmotic stress, growths of *P. marneffei* wild type, *atfA* mutant and *atfA* complemented strains on media supplemented with NaCl or sorbitol were not different at both 25°C and 37°C (Figure 6A to 6F).

### *atfA* expression is increased under heat shock condition but does not play a major role in heat stress response of *P. marneffei* conidia and is not regulated by SakA

**Figure 7:**
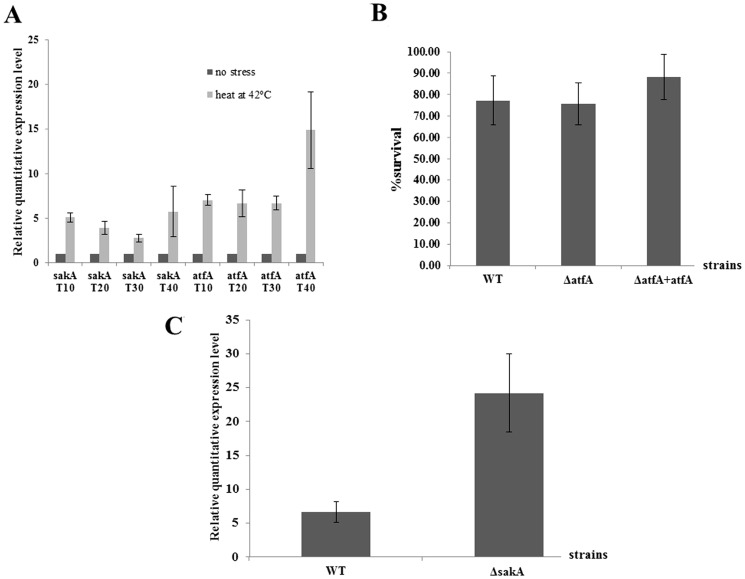
Gene expressions and survival of *P. marneffei* under heat stress at 42°C. (A) Relative RNA expression of *sakA* and *atfA* genes of conidia from *P. marneffei* wild type determined by real-time PCR. Conidia were incubated at 42°C for 10, 20, 30 or 40 minutes. Total RNA was extracted from conidia and subjected to real-time PCR. Expression level of heat stress cells is presented as relative value to the expression level from no stress cells which is given a value of 1. GAPDH gene expression level was used to normalize amounts of input RNA. (B) Survival of conidia from *P. marneffei* wild type (WT), *atfA* mutant (Δ*atfA*) and complemented strains (Δ*atfA* + *atfA*) after incubating in BHI at 42°C for one hour. (C) Relative RNA expression level of *atfA* gene of conidia from *P. marneffei* wild type (WT), *sakA* mutant (Δ*sakA*). Conidia were incubated at 42°C for 20 minutes and total RNA was extracted and subject to real-time PCR. Results were obtained from three independent experiments and standard error bars of the mean bars are shown (p<0.05).

In a previous study, the functions of the stress-activated kinase A (*sakA*) gene of *P. marneffei* were identified. The results demonstrated that *sakA* gene played a role in oxidative and heat stress responses of conidia and the yeast cell production at 37°C in *P. marneffei* (unpublished data). In this study, expressions of *sakA* and *atfA* genes in conidia of *P. marneffei* wild type incubated at 42°C for 10, 20, 30 and 40 minutes were evaluated. The results revealed that the expressions of both genes were increased at every time point (Figure 7A). However, growth of conidia from *atfA* mutant at 39°C was similar to the wild type, and *atfA* complemented strains (Figure 6G) and viabilities of all strains were not significantly different when the temperature was increased to 42°C (Figure 7B). To investigate whether SakA regulates the expression of *atfA* gene under heat stress at 42°C, conidia of *sakA* mutant were incubated at 42°C for 20 minutes and the relative expression level of *atfA* gene were identified. The result demonstrated that deletion of *sakA* gene did not affect the increase of *atfA* expression under heat shock stress. On the other hand, the expression of *atfA* in *sakA* mutant was significantly higher than the wild type strain under this kind of stress (Figure 7C).

### *atfA* gene participates in stability of cell membrane but is not involved in chitin deposition and response to cell wall stress

**Figure 8:**
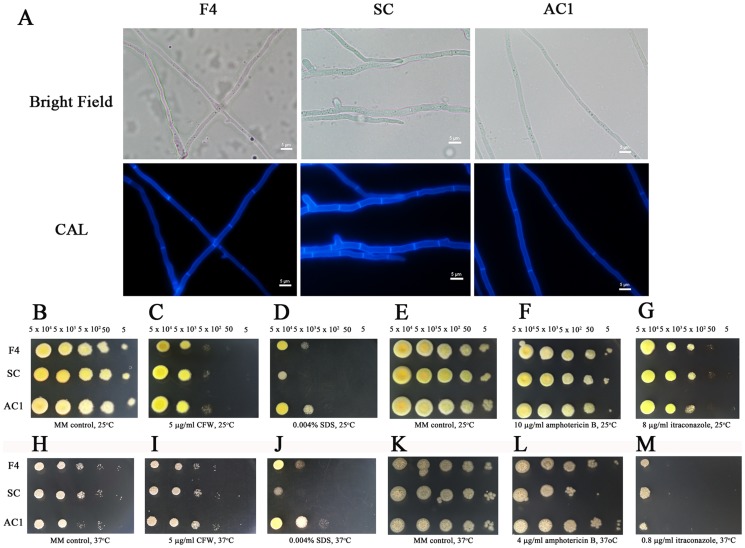
Deletion of *atfA* does not affect chitin deposition and cell wall integrity. (A) *P. marneffei* wild type (F4), *atfA* mutant (SC) and *atfA* complemented (AC1) strains were grown for seven day at 25°C on PDA and stained with CFW day four and day seven to visualize cell wall and septa. Scale bar represents five micrometers. (B to M) five microliters of cell dilutions (5×10^4^ to 5 cells) of wild type, *atfA* mutant and *atfA* complemented strains were inoculated on media supplemented with 5 µg/ml CFW(C and I), 0.004% SDS (D and J), 10 µg/ml and 4 µg/ml amphotericin B (F and L) and 8 µg/ml and 0.8 µg/ml itraconazole (G and M). (B and E) and (H and K) represent MM control plates at 25°C and 37°C, respectively.

To investigate the function of *P. marneffei atfA* in cell wall integrity, wild type, *atfA* mutant and *atfA* complemented strains were grown on PDA at 25°C and the cells were stained with CFW (an anionic dye that binds to nascent chitin chain) after four and seven days of incubation to visualize cell wall and chitin deposition. The results demonstrated that all strains showed normal chitin deposition along their hyphae (Figure 8A). In addition, the response of conidia from the *atfA* mutant to cell wall disrupted agent CFW was similar to those of wild type and *atfA* complemented strains (Figure 8C and 8I). This suggested that *atfA* gene may not play any role in chitin deposition and cell wall integrity of *P. marneffei*. For the role of *atfA* gene on cell membrane stability, all strains were grown on media containing membrane disrupting agent (SDS) [29] and antifungal agents (amphotericin B and itraconazole). The results showed the sensitivity of the mutant to SDS at both 25°C and 37°C (Figure 8D and 8J). Only the sensitivity to amphotericin B was observed at 37°C when compared to the wild type and complemented strains (Figure 8L). Growth of all strains was not different in the presence of itraconazole at both 25°C and 37°C (Figure 8G and 8M).

### *sakA* and *atfA* genes are not required for response under UV stress but play a role in *P. marneffei* survival inside both mouse and human macrophages

**Figure 9:**
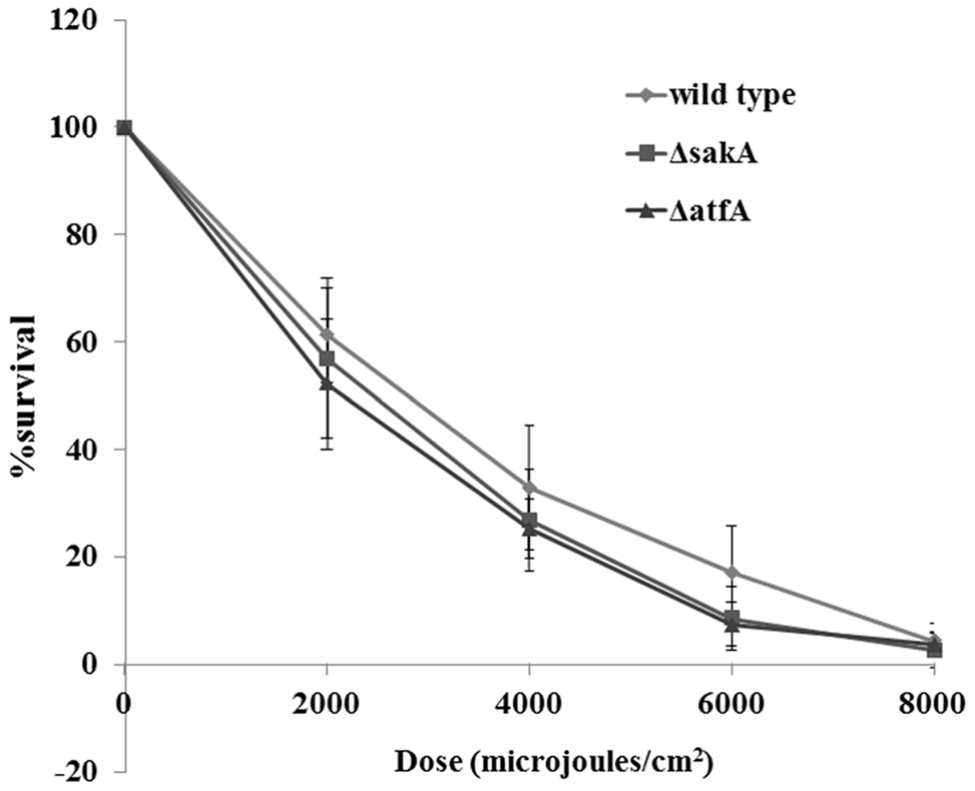
Susceptibility of conidia from *P. marneffei* wild type, *sakA* mutant (Δ*sakA*) and *atfA* mutant (Δ*atfA*) to UV light. Conidia of each strain were plated in duplicate on SDA and exposed to different UV light radiation at 0, 2000, 4000, 6000 and 8000 microjoules/cm^2^. Data are from three independent experiments and standard error bars of the mean bars are shown (p<0.05).

**Figure 10:**
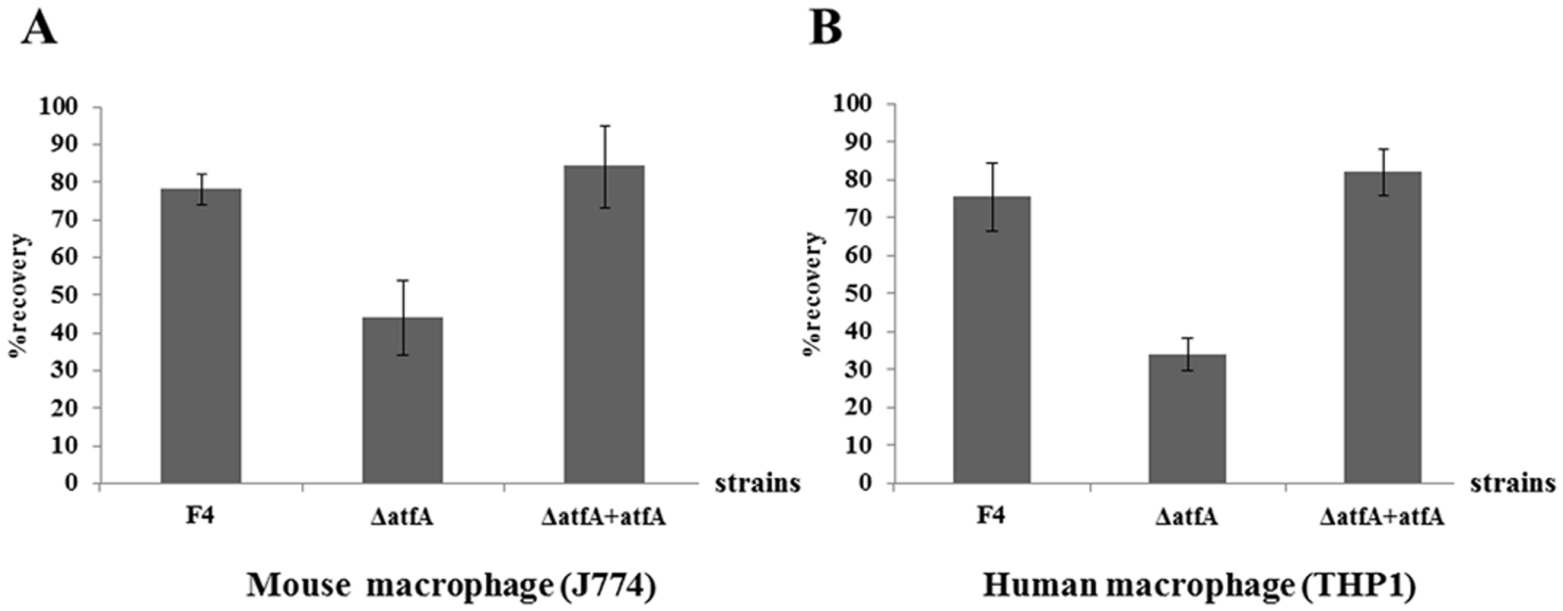
Survival of *P. marneffei* inside macrophage. Mouse (A) and human (B) macrophages were infected with conidia of *P. marneffei* wild type (F4), *atfA* mutant (Δ*atfA*) and *atfA* complemented (Δ*atfA* + *atfA*) strains. Percent recovery was calculated from number of colonies recovered after two hours and 24 hours post-infection. Data are from three independent experiments and standard error bars of the mean bars are shown (p<0.05).

To demonstrate the roles of *sakA* and *atfA* genes under UV stress, *P. marneffei* wild type, *sakA* and *atfA* mutant conidia were exposed to different doses of UV light. The results showed that the survival of all strains after exposure to UV light were not significantly different (Figure 9). Previous study, the role of *sakA* gene on survival of *P. marneffei* conidia inside macrophages was identified. The results showed that this gene is required for conidia to survive inside both mouse and human macrophages (unpublished data). In this study, to investigate the role of *atfA* gene for survival of *P. marneffei* inside macrophages, both mouse (J774) and human (THP1) macrophages were infected with conidia from *P. marneffei* wild type, *atfA* mutants, and *atfA* complemented strains. Twenty four hours post-infection, the survival of *atfA* mutants was significantly decreased in both cell types comparing to wild type and *atfA* complemented strains (Figure 10A and 10B).

## Discussion

In this study, we have shown that *P. marneffei atfA* encodes a protein in a bZip transcription factor family and plays a role in oxidative stress response and survival inside macrophages of the conidia. Responses to environmental stress are significant factors for many pathogenic fungi to survive outside and inside host cells and establish the disease. In this study, sensitivity of the conidia isolated from *P. marneffei atfA* mutant to osmotic stresses (NaCl and sorbitol), and cell wall stress (CFW) are similar to those of wild type and complemented strains. These results suggest that *P. marneffei atfA* might be involved in specific stress responses other than the highly osmotic and the cell wall stress responses. For osmotic stress response, *P. marneffei atfA* mutant seemed to tolerate both NaCl and sorbitol better than the wild type and complemented strains. This might occur from compensation of the SakA pathway homolog. It has been shown that *A. nidulans* possesses two functional Hog1-type MAPKs including SakA/HogA and MpkC [30]. Similar to HogA, MpkC can be phosphorylated by the MAKK protein (PbsB) and overexpression of *mpkC* gene can inhibit the high susceptibility to osmotic stress of *A. nidulans hogA* mutant [30], [31]. In *A. fumigatus*, two Hog1 orthologues, SakA and MpkC participate in response to oxidative and nutritional stresses, respectively [32]. In addition, *A. fumigatus sakA* also shares a conserved role in osmotic stress response as in *S. cerevisiae* such that *A. fumigatus sakA* controls the transcription of protein DprB required for osmotic and pH stress [33]. This indicates that overcompensation of *P. marneffei atfA* mutant strain to osmotic stress might come from the activation of the stress signaling pathway or transcription factor other than SakA-AtfA pathway.

Under stress from SDS, a membrane perturbation agent and antifungal agent amphotericin B, a polyene which irreversibly binds to ergosterol resulting in disruption of fungal membrane integrity and cell death, survival of conidia of the mutant is less than those of the wild type and complemented strains (at both 25°C and 37°C for SDS and 37°C for amphotericin B). This indicates the participation of this gene in cell membrane integrity. For heat stress response, it reveals that at 42°C, the mRNA expression level of *atfA* gene in *P. marneffei* conidia is significantly increased in both wild type and *sakA* mutant strains. This suggests that heat shock stress might activate the expression of this gene independently from *sakA*. One possibility is that there is a crosstalk between the SakA pathway and another MAP kinase pathway in *P. marneffei* that is activated under heat stress and this MAPK pathway can stimulate the expression of *atfA* gene in the absence of *sakA*. In yeast *S. cerevisiae*, many stress conditions including low pH, hyperosmotic, oxidative and heat stresses can activate both Hog1 and cell wall integrity (Slt2/Mpk1) pathways [34]. However, deletion of *atfA* gene does not affect the susceptibility to heat stress at both 39°C and 42°C. Thus, the heat shock stress could activate the expression of *atfA* gene, but *atfA* gene does not play a major role in heat stress response in *P. marneffei*. In *S. cerevisiae*, it has been shown that there is cross protection among different stressors. Heat shock transcription factor (HSF1), MSN2 and MSN4 transcription factors play a major role in heat shock stress response and heat shock can stimulate tolerance to oxidative and osmotic stresses [35]. In *A. nidulans*, AtfA plays a role in response against oxidative and heat stresses but not osmotic stress of conidia [8]. In *Aspergillus oryzae*, two genes encoding bZip type proteins similar to ATF/CREB, *atfA* and *atfB* have been reported [36]. AtfB reveals a short N-terminal region comparing to AtfA and play a role in heat stress response and development of conidia under high osmotic stress. Nevertheless, *atfB* homolog in *P. marneffei* has not been reported.

Reactive oxygen species (ROS) produced by host immune cells such as macrophages, neutrophils and other phagocytic cells are toxic to some fungal pathogens and are able to eliminate these pathogens from the host body [37]. Therefore, to protect themselves from host immunity, the stress response systems that can send the signal inside fungal cells to produce enzymes or molecules used to detoxify ROS are required. The results from this study demonstrated that *atfA* gene was involved in response to only organic hydroperoxide, *t*-BOOH but not for H_2_O_2_ and menadione. In addition, *P. marneffei atfA* mutant strain seemed to grow better than the wild type and complemented strains under H_2_O_2_ stress. This result indicated that *atfA* gene did not play a major role in oxidative stress response and *P. marneffei* might use different transcription factors to sense different ROS. *P. marneffei* possesses, Skn7 functioned as transcription factor and response regulator, that is involved in response to H_2_O_2_ [16]. In other fungi such as *S. cereivsiae* and *Alternaria alternata*, Skn7 was also found to regulate the expressions of genes in response to H_2_O_2_ and *t*-BOOH stress [38]–[40]. H_2_O_2_ is a byproduct of aerobic aspiration and widely used as a model for oxidative stress condition. This molecule can be detoxified to H_2_O and O_2_ by catalase. *t*-BOOH is a simple organic alkylhydroperoxide that is frequently used to generate lipid oxidation. Glutathione peroxidase is used to reduce this toxic substance but not catalase [35], [41]. It has been shown that cellular signaling response of budding yeast *S. cerevisiae* activated by H_2_O_2_ is different from that is induced by *t*-BOOH [35], [42]. Whereas Yap1, a bZip transcription factor of the AP-1 family plays a crucial role for tolerance to H_2_O_2_, diamide and cadmium in *S. cerevisiae*, the other transcription factor Cad1 is activated under *t*-BOOH treatment [35], [42]. In citus pathogen *A. alternata* AP1 is associated with the detoxification of ROS and pathogenesis [40]. Further study should be done to investigate the effect of *atfA* gene on transcription of glutathione peroxidase gene under *t*-BOOH stress.

Because the induction of the MAPK pathway results in the transcriptions of genes responding to environmental stresses, it is interesting to understand the relationship between the MAPK protein and the transcription factor inside the nucleus. The functional analysis of *P. marneffei sakA* (*hog1* homolog) was performed and the results showed that this gene participated not only in heat stress response and oxidative tolerance to H_2_O_2_ and *t*-BOOH of the conidia but also involved in asexual development, yeast cell production at 37°C, and chitin deposition along the hyphae (unpublished data). In this study, the susceptibilities of different strains of *P. marneffei* to murine and human macrophages were done. Both *sakA* and *atfA* mutants were more susceptible to these phagocytic cells comparing to wild type and complemented strains indicating the involvement of *sakA* and *atfA* genes in survival of *P. marneffei* conidia inside macrophages. However, the result from macrophage infection experiment was not correlated with oxidative susceptibility test. In *P. marneffei*, it has been shown that after phagocytosis by macrophages of immunocompetent host, the fungus are cleared via nitric oxide (NO) which is stimulated by T-cell derived IFN-γ [43]. Therefore, *P. marneffei atfA* might be involved in response against RNS generated by host macrophages rather than ROS. The outcomes from this study showed that *atfA* gene participated in a part of the systems regulated by *sakA* gene including tolerance to hydroperoxide (*t*-BOOH) and survival inside macrophages. This indicates that *sakA* might interact with other MAPK proteins or other transcription factors to control gene expressions that are not dependent on the *atfA*. In *S. pombe*, the Sty1/Wis1 pathway is involved in osmotic, oxidative and heat stress responses and the control of mitotic initiation. It has been shown that *S. pombe* Atf1 directly binds and is phosphorylated by the Sty1 MAP kinase under these stress conditions. However, deletion of *atf1* did not have any effect on the timing of mitotic initiation [44].

It has been shown that the regulation of ATF function is conserved. In mammalian, transcription factor ATF-2 is controlled by SAPK pathway similar to Atf1 of fission yeast *S. pombe* and AtfA of *A. nidulans* [8], [44]. The results from this study and previous study demonstrated that AtfA of *P. marneffei* might play a role downstream of SakA signaling pathway under certain stresses (SDS, *t*-BOOH and macrophage infection). However, further study on the interaction between SakA and AtfA under these stress conditions should be performed to help us understand more clearly the stress signaling pathways in dimorphic fungi.
